# Impact of Repeated Variant Exposures on Cellular and Humoral Immunogenicity Induced by SARS-CoV-2 Vaccines †

**DOI:** 10.3390/vaccines12121408

**Published:** 2024-12-13

**Authors:** Leire Fernández-Ciriza, José Luis del Pozo, Nazaret Betanzos, Álvaro González, Alejandro Fernandez-Montero, Francisco Carmona-Torre, Marta Vidaurreta, Silvia Carlos, Gabriel Reina

**Affiliations:** 1 Department of Microbiology, Clínica Universidad de Navarra, 31008 Pamplona, Spain; lfciriza@unav.es (L.F.-C.); nbetanzos@unav.es (N.B.); mvidaurreta@unav.es (M.V.); gabi@unav.es (G.R.); 2 Navarra Institute for Health Research (IdiSNA), 31008 Pamplona, Spain; agonzaleh@unav.es (Á.G.); afmontero@unav.es (A.F.-M.); fcdelatorre@unav.es (F.C.-T.); scarlos@unav.es (S.C.); 3 Infectious Diseases Division, Clínica Universidad de Navarra, 31008 Pamplona, Spain; 4 Department of Biochemistry, Clínica Universidad de Navarra, 31008 Pamplona, Spain; 5 Department of Occupational Medicine, Universidad de Navarra, 31008 Pamplona, Spain; 6 Department of Preventive Medicine and Public Health, Universidad de Navarra, 31008 Pamplona, Spain

**Keywords:** mRNA vaccine, Omicron, reinfection, cellular immunity, humoral immunity, fourth dose, COVID-19

## Abstract

Background/Objectives: The emergence of the Omicron variant has complicated COVID-19 control and prompted vaccine updates. Recent studies have shown that a fourth dose significantly protects against infection and severe disease, though long-term immunity data remain limited. This study aimed to assess Anti-S-RBD antibodies and interferon-γ levels in healthcare workers 12 months after receiving bivalent Original/Omicron BA.4-5 fourth SARS-CoV-2 vaccine. Methods: In this prospective cohort study, 549 healthcare workers were categorized by the initial vaccination schedule, with 229 individuals having received the fourth SARS-CoV-2 vaccine dose. Blood samples were collected from all participants 12 months post-vaccination. Results: Significant differences in Anti-S-RBD antibody levels were observed between those receiving a fourth dose and those who did not, while no differences were seen in interferon-γ levels. After 12 months, there were no significant differences in humoral and cellular immunity response between volunteers primoinfected or reinfected across different periods by the Omicron variant. A multivariable analysis revealed an association between high antibody levels (>6000 U/mL) and interferon-γ levels (OR: 3.13; 95% CI: 1.3–7.7; *p* < 0.05). Regarding primary vaccine schedules, participants vaccinated with ChAdOx1 (a single or double dose) had notably lower antibody levels compared to those who received mRNA-based vaccines. Additionally, the study shows a higher frequency of multiple infections among those with a single-dose ChAdOx1 primary schedule (OR: 6.24; 95% CI: 1.25–31.15; *p* < 0.01). Conclusions: Overall, mRNA-based vaccines exhibited stronger long-term immunogenicity. Repeated exposure to the Omicron variant seems to mitigate immune imprinting from the wild-type SARS-CoV-2. An association was observed between high antibody levels and a strong cellular response, although the correlation was not linear.

## 1. Introduction

Since the emergence of the first SARS-CoV-2 variant of concern (VOC) in late 2020, new variants with increased transmissibility and immune evasion have presented ongoing challenges for COVID-19 control efforts. By October 2021, the Delta variant was overtaken by Omicron (B.1.1.529), which quickly dominated global cases. Over 100,000 Omicron genomes were sequenced within months [1], with prevalent subvariants including BA.1, BA.2, BA.4, and BA.5 [2].

A retrospective study in Qatar during the Omicron wave found that the BNT162b2 booster reduced symptomatic infections by 86.1% for Delta but only 49.4% for Omicron [3], while a similar study in England showed that booster protection against mild infection waned over weeks, especially for Omicron [4]. These declines accelerated efforts to update vaccination strategies, and by October 2022, the European Union authorized four new vaccines. These new vaccines targeted the Original variant (wild-type (WT)) as well as the Omicron variants BA.1, BA.4, and BA.5. The four approved vaccines were combined as follows: Comirnaty Bivalent Original/Omicron BA.1, Spikevax Bivalent Original/Omicron BA.1, Comirnaty Bivalent Original/Omicron BA.4-5, and Spikevax Bivalent Original/Omicron BA.4-5 [5,6,7]. In a prospective cohort study involving 32,542 individuals, the effectiveness of the bivalent (Original/Omicron BA.1) mRNA booster vaccination against Omicron infection was assessed. The study found that vaccination provided an overall relative vaccine effectiveness of 31% in individuals aged 18–59 years and 14% in those aged 60–85 years. This analysis was conducted among individuals who had previously completed the primary vaccination series and received at least one booster dose, with adjustments made for prior infection history [8]. In a study conducted in the United States between August 2022 and February 2023 using a nationwide dataset, the relative vaccine effectiveness (rVE) of two mRNA bivalent (Original/Omicron BA.4/BA.5) vaccines, mRNA-1273.222 (Moderna) and BNT162b2 (Pfizer-BioNTech), was evaluated. The rVE was assessed against COVID-19-related hospitalizations and outpatient visits among 1,034,538 recipients of mRNA-1273.222 and 1,670,666 recipients of BNT162b2. The adjusted rVE was found to be 9.8% (95% CI: 2.6–16.4%) and 5.1% (95% CI: 3.2–6.9%) for mRNA-1273.222 and BNT162b2, respectively [9]. In Spain, the fall–winter 2022 campaign focused on these adapted vaccines, prioritizing vulnerable populations and healthcare workers [10].

Recent studies have indicated that a fourth vaccine dose offers significant protection against Omicron-related infections and severe disease. For example, an Israeli study reported a twofold decrease in infections and a threefold decrease in severe cases within four weeks of the fourth dose [11]. Medium-term follow-up studies further support these findings [12]. However, long-term data (beyond 12 months) on humoral and cellular immunity post-fourth dose are limited, as is evidence on how previous infections or the primary vaccination schedule influence long-term Anti-S-RBD and interferon-γ responses.

The objective of this study was to assess levels of Anti-S-RBD antibodies and interferon-γ in healthcare workers (HCW) one year after receiving a fourth dose of the SARS-CoV-2 vaccine. Additionally, we examined the impact of primary infections and reinfections occurring at different pandemic phases, as well as the influence of initial vaccination regimens on these immune responses.

## 2. Materials and Methods

### 2.1. Study Design and Participants

A longitudinal analysis was conducted within a prospective cohort study started in March 2021 (SARS-CoV-2 Vaccine Immunity Navarra-SAVIN-Project) at Clinica Universidad de Navarra (Pamplona, Spain). To be enrolled as a volunteer in the study, inclusion criteria included HCWs at this hospital who received full primary vaccination coverage against SARS-CoV-2 (enrolment period: March 2021). Exclusion criteria included pregnancy, breastfeeding, oncology patients, and immunocompromised individuals. The current analysis was performed on samples collected from adult volunteers in October 2023, 12 months after receiving the fourth vaccine dose against SARS-CoV-2 (October 2022). Both previously SARS-CoV-2-infected subjects and naïve subjects were able to participate.

**Figure 1:**
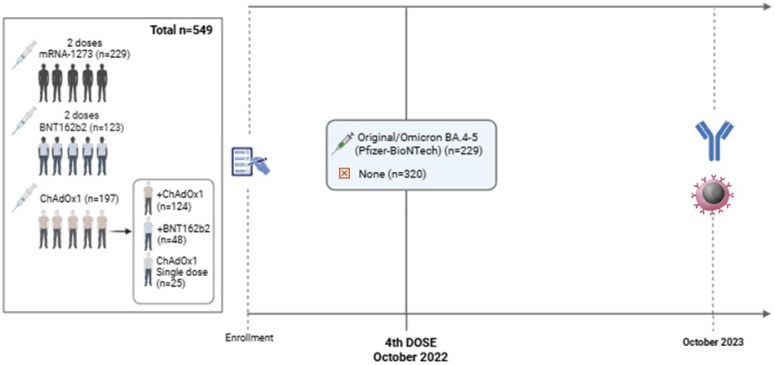
Flowchart of SAVIN participants. Recruitment and follow-up summary for humoral and cellular response evaluation after booster dose (Created in BioRender.com).

Five hundred and forty-nine subjects were enrolled in the analysis (out of 709 individuals initially enrolled in the SAVIN study) [13]. They were classified depending on their primary vaccination schedule (group 1: mRNA-1273 vaccine; group 2: BNT162b2 vaccine; groups 3/4/5: ChAdOx1 nCoV-19 as a first-dose vaccine). For mRNA-1273 and BNT162b2, the vaccination schedule included 2 doses for all participants (previously infected and non-infected individuals). For those who received a first dose of the ChAdOx1 vaccine and had not been previously infected, they received a second dose of the ChAdOx1 (group 3) or BNT162b2 (group 4) vaccine at free choice. Individuals previously infected and vaccinated with a first dose of the ChAdOx1 vaccine did not receive any second dose (group 5) (Figure 1). Enrolment details and the proportion of participants within this cohort can be found elsewhere [13].

Within the 549 fully vaccinated participants (primary schedule), in October 2022, 229 volunteers received the fourth dose of the COVID-19 vaccination campaign of Comirnaty Original/Omicron BA.4-5 (Pfizer-BioNTech) 12 months before sample collection (12 months of follow-up, single point) (Figure 1). Plasma samples were collected from all participants (n = 549) with the aim of studying humoral immunity (Anti-S-RBD levels), while 174 whole blood samples were collected to study cell-mediated immunity (interferon-γ levels (IFN-γ)). To study primoinfections and reinfections, individuals with prior infections or not were classified by measuring Anti-N antibodies (antibodies produced by natural infection) at eight follow-up points since the beginning of the study (from March 2021 to October 2023). To identify the variants responsible for infections in each study period, the start and end dates of each period were considered, and the predominant variants were inferred by observing which ones were most prevalent during each period [14,15,16]. Participants’ data were recorded from the hospital register.

### 2.2. Humoral Response Evaluation

Two distinct commercial chemiluminescence assays were used for the detection of antibodies against SARS-CoV-2. Quantitative detection of total antibodies against the receptor binding domain (RBD) of the SARS-CoV-2 spike protein (S) and qualitative detection of total antibodies against the viral nucleocapsid (Anti-N) were performed using Elecsys^®^ reagents (Anti-SARS-CoV-2-S and Anti-SARS-CoV-2 test. Roche Diagnostics, Penzberg, Germany). Both tests use the Wuhan WT variant as the antigen [17]. The quantification of Anti-S-RBD levels, indicative of humoral responses induced by either vaccination or SARS-CoV-2 infection, was conducted using a cutoff value of 0.8 U/mL, with quantification ranging up to 200,000 U/mL. Anti-N reactivity, reflecting prior natural infection, was determined by a cutoff index (COI) of 0.150, as previously suggested, as this is a follow-up study [13]. The official COI for Anti-N provided by the manufacturer is 1.0. Primary infections and reinfections were recorded considering the Anti-N data obtained in samples collected over 8 monitoring points between March 2021 and October 2023.

### 2.3. Cellular Response Evaluation

The cellular immune response to SARS-CoV-2 was studied by measuring interferon-gamma (IFN-γ) production by stimulated CD4+ and CD8+ lymphocytes using the WT variant in heparinized whole blood (Covi-FERON Tubes, SD-BIOSENSOR^®^, Cheongju-si, Chungcheongbuk-do, Republic of Korea; or SARS-CoV-2 IGRA Test, Euroimmun^®^, Lübeck, Germany). Blood samples were processed within 8 h of venipuncture. To carry out the test, 1 mL of blood was inoculated into SARS-CoV-2 S protein-sensitized tubes. Following inoculation, the tubes were incubated for 16–24 h at 37 °C, followed by the measurement of IFN-γ levels by an enzyme-linked immunosorbent assay (ELISA Euroimmun^®^, Germany). Samples were considered non-reactive when IFN-γ production following antigen stimulation was below 250 mIU/mL and reactive when IFN-γ production was equal to or above 250 mIU/mL. Cellular immunity was considered weak or strong depending on the concentration of IFN-γ. Low levels ranged from 250–1499 mIU/mL, while high levels were defined as greater than or equal to 1500 mIU/mL.

### 2.4. Statistical Analysis

Due to their non-normal distribution determined with the Shapiro–Wilks test, concentrations were expressed as the median and interquartile range (IQR), and the non-parametric Mann–Whitney U test was applied to assess the statistical differences in antibody levels. Spearman’s correlation test for non-parametric variables was performed to assess the correlation between antibody levels and IFN-γ. Odds ratios were calculated by multivariable logistic regression to determine the strength of the association between Anti-S-RBD antibody levels and IFN-γ and between the ChAdOx1 vaccine and the number of infections experienced. The analysis was adjusted by sex, age, vaccine schedule, and inoculation of 4th dose. Some multiple linear regression analyses of Anti-S-RBD levels were conducted, adjusted for age and sex, to investigate the association between different initial vaccination schedules and various scenarios of primoinfection or reinfection, the number of previous infections, and the number of boosters received.

Statistics and graphs were obtained using Stata 14.0. A two-tailed *p*-value < 0.05 was considered statistically significant.

## 3. Results

### 3.1. Description of Participants After 12 Months of Follow-Up According to the Fourth Dose Inoculation

All participants were classified based on whether they had received a fourth dose of the SARS-CoV-2 vaccine. Overall, 88% of participants were women with a mean age of 48.3 ± 10.2 years, and 98% had experienced a SARS-CoV-2 infection (60.5% had been infected once, while 37.9% had been infected two or more times). Table 1 presents the humoral and cellular immune responses 12 months after the fourth dose, comparing individuals with prior infections to those without.

All participants showed antibody production and 97.1% of the participants had a positive cellular response. Anti-S-RBD antibody and IFN-γ levels were studied among previously infected volunteers to assess the difference between those who received the fourth dose of the vaccine and those who did not. Statistically significant differences were found in antibody levels (*p* < 0.05) while none were found in IFN-γ levels (*p* = 0.3). Naïve volunteers were not analyzed due to the small sample size (n = 10 in the humoral immunity group and 8 in the cellular immunity group).

Differences between previously infected and non-infected volunteers also were studied. Anti-S-RBD levels were significantly higher among previously infected volunteers in contrast to those who had not been infected in the total number of participants (regardless of whether or not they had received the fourth dose) (*p* < 0.05). There are no statistically significant differences in IFN-γ levels between the total number of infected and non-infected volunteers (*p* = 0.4). The same pattern was observed among volunteers boosted with the fourth dose (*p* < 0.05 humoral immunity; *p* = 0.8 cellular immunity).

### 3.2. Humoral Immunity 12 Months After Fourth Dose Inoculation

**Figure 2:**
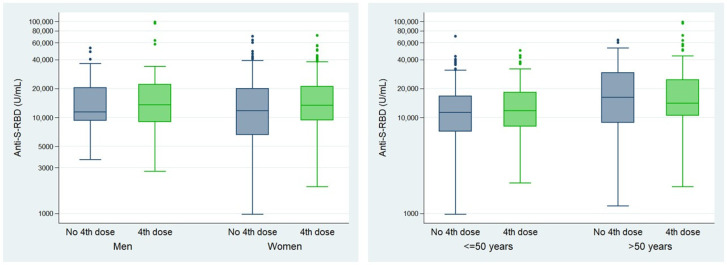
Humoral immune response (Anti-S-RBD (U/mL) median (IQR)) by age and sex (n = 549). Statistically significant difference: ** (<0.01). The Mann–Whitney U test was used to calculate *p*-values.

The humoral response was analyzed in volunteers who were previously vaccinated and not with the October 2022 fourth dose after 12 months of follow-up. The influence of sex and age was studied. The results are presented in Figure 2 (Table S1).

After 12 months of follow-up, Anti-S-RBD levels did not differ based on sex (*p* = 0.23). However, they were significantly higher among volunteers over the age of 50 (*p* < 0.01) (Figure 2). This difference was not attributable to the number of infections in each age group (equal or below 50 or over 50 years), as the percentage of participants with one or two infections was very similar: 60.7–60.3% and 34.7–35.8%, respectively.

The hybrid immunity at different follow-up points was also studied. Antibody levels were analyzed in participants who received the fourth dose after 12 months of follow-up. *p*-values were calculated considering volunteers with primoinfections and reinfections during different time periods (January 2022, January–June 2022, and July 2022–October 2023) to analyze potential differences in antibody levels between these groups. The predominant circulating variants during those periods were taken into account (Table 2). As explained above, primary infections and reinfections were established according to the evolution of Anti-N index levels.

No statistically significant differences were found between those who had been primoinfected or reinfected in January 2022, between January and June 2022, or between July 2022 and October 2023 (*p* = 0.8, *p* = 0.4, and *p* = 0.1, respectively). An adjusted linear regression analysis conducted at this stage concluded that there were no statistically significant differences between individuals vaccinated with different primary schedules in the groups of primoinfection and reinfection between July 2022 and October 2023, except for the ChAdOx1/ChAdOx1 regimen in the primoinfected group, which produced a lower response (*p* = 0.008).

Anti-S-RBD antibody levels were calculated based on the number of previous infections among the volunteers who received the fourth dose 12 months before (Table 3). Statistically significant differences were found between the group of uninfected patients and the groups of patients who had experienced an infection (one infection (*p* < 0.01) and two or more infections (*p* < 0.01)). However, no statistically significant differences were observed between participants who had been infected once and those who had been infected two or more times (*p* = 0.3).

### 3.3. Cellular Immunity 12 Months After Fourth Dose Inoculation

**Figure 3:**
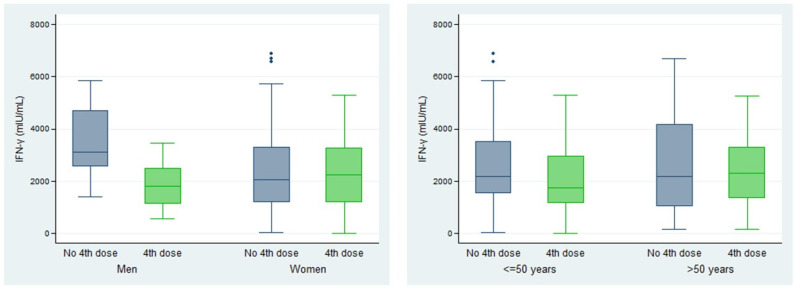
Cellular immune response (IFN-γ (mIU/mL) median (IQR)) by age and sex (n = 174). Statistically significant difference: *** (<0.01)). The Mann–Whitney U test was used to calculate *p*-values.

The cellular response was analyzed 12 months after the fourth dose in 174 volunteers. The influence of sex and age was studied. The results are shown in Figure 3 (Table S2).

No statistically significant differences were found between men and women, nor among the different age groups (under and over 50 years) (*p*-values for the total of volunteers: 0.18 and 0.97, respectively (Table S2)).

The effect of past primoinfections and reinfections on IFN-γ levels was studied in volunteers vaccinated in October 2022 after 12 months of follow-up, as in the humoral response. *p*-values were calculated considering volunteers with primoinfections and reinfections during different time periods (January 2022, January–June 2022, and July 2022–October 2023) to analyze potential differences in IFN-γ levels between these groups. The predominant circulating variants during those periods were taken into account (Table 4). Primary infections and reinfections were established according to the evolution of Anti-N index levels.

No statistically significant differences were found between those who had been primoinfected and reinfected in January 2022, between January and June 2022, or between July 2022 and October 2023 (*p* = 0.1, *p* = 0.3, and *p* = 0.5, respectively).

IFN-γ levels were calculated based on the number of previous infections among the volunteers who received a dose 12 months earlier (Table 3). No statistically significant differences were found between naïve and previously infected volunteers, neither in the group with one infection (*p* = 0.32) nor in the group of reinfections (*p* = 0.45). However, differences were found between those infected only once and those reinfected (*p* < 0.05).

Table 5 shows the medians (IQR) of Anti-S levels within those interferon ranges. An association was found, approaching the threshold of statistical significance (*p* = 0.05).

Similarly, a multivariable analysis was conducted to assess the association between high Anti-S-RBD levels (greater than 6000 U/mL; arbitrarily established) and IFN-γ levels, revealing a statistically significant association (OR: 3.13; IC95% 1.3–7.7; *p* < 0.05). Additionally, linear correlation tests were performed, showing no linearity between antibody levels and IFN-γ.

### 3.4. Immune Response According to Initial Vaccine Schedule 12 Months After Fourth Dose of Inoculation

The humoral and cellular immune responses were analyzed between all participants considering the initial vaccination schedule: mRNA-1273/mRNA-1273, BNT162b2/BNT162b2, ChAdOx1/BNT162b2, ChAdOx1/ChAdOx1, and ChAdOx1 single doses. The results are presented in Table 6.

After 12 months of follow-up, those who received ChAdOx1 single dose and ChAdOx1/ChAdOx1 as initial schedules showed lower antibody levels compared to those with schedules that included at least one dose of an mRNA vaccine (mRNA-1273/mRNA-1273 and BNT162b2/BNT162b2 *p* < 0.001, ChAdOx1/BNT162b2 *p* < 0.01). An adjusted linear regression analysis conducted at this stage concluded that there were no statistically significant differences between individuals with a different number of infections or number of booster doses among individuals who received mRNA-based primary schedules. In fact, statistically significant differences were found in the ChAdOx1/ChAdOx1 and ChAdOx1 single-dose regimens, resulting in lower antibody levels (*p* < 0.001). This is not the case for cellular immunity, where no significant differences in IFN-γ levels were observed (Table 6).

Additionally, the multivariable analysis demonstrated that the frequency of having two or more infections is higher among those volunteers who initially received the ChAdOx1 single-dose schedule (OR: 6.24, IC95%: 1.25–31.15); *p* < 0.01).

## 4. Discussion

Our study shows a high level of humoral and cellular immunity among healthcare workers vaccinated or not with a fourth dose of the SARS-CoV-2 vaccine after a long period of follow-up. This is consistent with the findings of Hyun et al., who conducted a 9-month follow-up study and concluded that the humoral immunity induced by the bivalent BA.4/5 mRNA COVID-19 vaccination persists for up to 9 months. However, cellular immunity was only monitored for 4 weeks [18]. Long-term surveillance studies are needed to describe the kinetics of cellular immunity.

Our findings suggest that mRNA-based vaccines demonstrate stronger long-term immunogenicity, particularly evident in antibody levels among individuals who received mRNA vaccines as part of their primary series, even 12 months after the fourth dose. In the Com-COV2 trial, geometric mean concentrations (GMCs) of anti-S antibodies were significantly higher at day 196 in participants with at least one mRNA-based vaccine in their schedules, as compared to those without. The study also found that anti-spike IgG levels declined more slowly for the ChAdOx1/ChAdOx1 schedule compared to ChAdOx1/mRNA-1273 and ChAdOx1/NVX-CoV2373 until day 112, after which waning rates became similar. A similar pattern was observed for BNT162b2/NVX-CoV2373 versus BNT162b2/BNT162b2 until day 56, with no significant differences in waning rates between homologous mRNA schedules over time [19]. These patterns align with our findings, supporting that mRNA vaccines sustain higher long-term antibody levels, as observed in our one-year follow-up and in Orlandi et al. [20]. Additionally, an association was found between the initial ChAdOx1 single-dose schedule and having suffered two or more SARS-CoV-2 infections, highlighting the importance of boosting within this population.

For cellular immunity, waning slows over time for all schedules, and after 4 months, T-cell levels plateau across regimens, according to a previous study [19]. This may explain the lack of significant differences in IFN-γ levels in our study over time. Further research confirms similar cellular responses from diverse vaccine technologies [21]. Supporting these trends, Goel et al. found that antibody waning rates decreased after day 89, with CD4+ T cell stability observed from 3 to 6 months, suggesting robust CD4+ T cell responses up to six months post-vaccination [22].

Our follow-up demonstrates that repeated exposure to the Omicron variant reduces significantly differences in antibody levels between primoinfected and reinfected individuals vaccinated with the WT variant as the primary schedule (as occurred following the administration of the third dose [23]). This phenomenon may be related to the mitigation of immune imprinting from the WT SARS-CoV-2 variant. This phenomenon is also observed in cellular immunity, in which no significant differences are shown between primoinfected and reinfected individuals. Consistent with Yisimayi et al. [24], who investigated immune imprinting dynamics post-WT primary vaccination, repeated Omicron infections increased neutralizing antibody values against Omicron more than a single breakthrough infection (BTI). Unvaccinated individuals with BTIs showed the highest neutralizing antibody values against Omicron, indicating that vaccination impacts immune imprinting, which may differ between mRNA and inactivated virus vaccines [24]. The previously mentioned study by Hyun et al. demonstrated that the bivalent BA.4/5 COVID-19 vaccine enhanced cross-neutralizing activity against the Omicron subvariants (BA.5, BQ.1.1, BN.1, and XBB.1), thus reinforcing the idea that immune imprinting may be overcome due to cross-reactive responses to different variants [18]. Similarly, cellular immunity showed broad cross-reactivity among variants [18], including WA1/2020, Delta, and Omicron [21]. After eight months, both CD8+ and CD4+ T cell responses to two different vaccine types (Ad.26.COV.2 and BNT162b2) maintained this cross-reactivity, supporting robust cellular immunity across SARS-CoV-2 variants [21].

Unexpectedly, participants over 50 years of age exhibited significantly higher antibody levels 12 months post-fourth dose, regardless of whether the dose was administered in October 2022. This trend of robust antibody production in older adults aligns with previous findings. In a Spanish study evaluating mRNA booster effectiveness in those aged 40 and older post-Omicron emergence, effectiveness estimates were higher among those aged 60–79 (58.0% [95% CI 55.8–60.4]) compared to individuals aged 40–59 years (49.9% [48.6–51.3]) [25]. Evidence also suggests that neutralizing and binding antibody levels are inversely related to COVID-19 risk and directly correlate with vaccine efficacy [26]. It is widely recognized that older age is generally associated with reduced antibody production, as evidenced by this study where volunteers with a median age of 79 had 2.5 times lower anti-RBD IgG levels than those aged 41. However, this difference decreased with additional vaccine doses (one versus two doses). Antibody levels also decline over time [27]. The elevated antibody levels seen in participants over 50 years may be due to dose count, time since vaccination, and lower age cutoff for group division in our study. In contrast, cellular immunity was unaffected by these factors, with parameters like age, booster interval, and sex showing no significant impact on IFN-γ T cell responses [28].

Our findings suggest an association between high antibody levels and strong cellular response, although the relationship between Anti-S-RBD and IFN-γ levels was not linear. Hollstain et al. observed that from 2 weeks to 3 months after the second vaccination dose, the correlation between humoral and cellular responses diminished across all vaccination regimens. This loss of significance may stem from the greater capacity of antibody titers to rise compared to the cellular immune response, leading to a weaker association between the two [28]. Similarly, another study demonstrated that one year after the first dose and following a booster, no correlation was found between humoral and cellular responses in previously infected individuals, whereas such a correlation was maintained in naïve participants [29]. Given that 98.2% of our participants had prior infection, this factor likely influenced our results.

Our study has certain limitations. First, some participant groups had small sample sizes, which could affect the robustness of our conclusions. Second, there was only one follow-up point to monitor previous infections over the course of 2023, although we do have data from seven follow-up points between 2021 and 2022. Additionally, it was not possible to measure IFN-γ levels stimulated separately by the WT and the Omicron variant. However, it has been described that T lymphocytes cross-recognize different variants, as they can recognize a variety of epitopes in antigenic regions that are more conserved within the Spike protein [17]. Therefore, we believe that our results are not influenced by this limitation. Similarly, the antibodies detected to study humoral immunity were stimulated by antigens from the WT variant and not with variant-specific Spike proteins. Previous studies have shown that antibody detection techniques based on WT variant antigens may detect lower antibody levels in individuals infected with the Omicron variant who have not been previously vaccinated or infected. However, all our volunteers were previously vaccinated, and therefore, we understand that this phenomenon does not affect our findings [17,30]. Lastly, as an observational study, there is a risk of participation bias, as participants were self-selected from hospital staff. Despite these limitations, our study also has notable strengths. It is one of the few to analyze both humoral and cellular immunity simultaneously, and few studies have followed participants for as long as one year after their fourth dose of the SARS-CoV-2 vaccine and almost three years after the first vaccination schedule. Moreover, we accounted for several factors that influence vaccine immunogenicity, including primary vaccination schedules and infections/reinfections with different viral variants.

## 5. Conclusions

Overall, our findings provide valuable insights into the immune response dynamics following vaccination with a bivalent formulation containing antigens from both the WT and Omicron variants. Although our study focuses on the antibody response against the WT antigen, the results highlight the potential effects of immune imprinting from ancestral variants. Importantly, individuals who have not been vaccinated with mRNA-based vaccines may require additional attention in future vaccination campaigns. Further research is needed to directly compare responses to both WT and Omicron antigens to better elucidate the mechanisms and implications of immune imprinting and the use of mRNA technology in the long term.

## Figures and Tables

**Table 1:** Participants’ characteristics based on inoculation of the fourth vaccine dose.

	4th Dose	No 4th Dose	TOTAL
N	229	320	549
Age, mean (SD)	49.9 (9.8)	47.1 (10.4)	48.3 (10.2)
Women (%)	86.9	88.8	88
SARS-CoV-2 infections (%)	96.1	99.7	98.2
Anti-S-RBD (U/mL) median (IQR) 12 months after administration			
N	220 13,642 * (9328–21,521.5)	319 11,795 * (7185–20,195)	539 12,851 * (8383–20,405)
SARS-CoV-2 previously infected individuals			
N	9 5165 * (4264–11,693)	1 1385 (-)	10 4953 * (4227–11,693)
SARS-CoV-2 non-previously infected individuals			
IFN-γ (mIU/mL) median (IQR) 12 months after administration			
N	71 2166 (1208–3165)	95 2191 (1325–3970)	166 2187.5 (1323–3321)
SARS-CoV-2 infected individuals			
N	7 2363 (824–3429)	1 1130 (-)	8 1753.5 (977–2958)
SARS-CoV-2 non-infected individuals			

Statistically significant difference: * (<0.05). The Mann-Whitney U test was used to calculate *p*-values.

**Table 2:** Humoral immune response in non-infected, primarily infected, and reinfected individuals at different time points (only participants who received the fourth dose are included) (n = 229).

	Infecting Variant	N	Anti-S-RBD (U/mL) Median (IQR)	*p*-Value
Primoinfection before December 2021	Wuhan, Alpha, Delta [14,15]	3	17,078 (10,808–37,872)	-
Primoinfection January 2022	Omicron (BA.1) [14,15]	23	12,620 (8003–21,311)	NS
Reinfection January 2022	Omicron (BA.1) [14,15]	5	13,393 (10,263–14,029)	
Primoinfection January–June 2022	Omicron (BA.1, BA.2) [14,15]	69	13,115 (8991–20,442)	NS
Reinfection January–June 2022	Omicron (BA.1, BA.2) [14,15]	17	12,087 (6735–17,166)	
Primoinfection July 2022–October 2023	Omicron (BA.5, BQ.1, XBB.1.5) [16]	50	16,639.5 (10,302–32,872)	NS
Reinfection (1 or more) July 2022–October 2023	Omicron (BA.5, BQ.1, XBB.1.5) [16]	54	13,143 (9966–17,820)	
Non infected	-	8	4953 (4245.5–11,280)	-

NS: non-significant. The Mann–Whitney U test was used to calculate *p*-values.

**Table 3:** Humoral and cellular immunity response according to the number of infections (only individuals receiving fourth dose are shown) (n = 229).

Number of Infections	Humoral Response (N)	Anti-S-RBD (U/mL) Median (IQR)	Cellular Response (N)	IFN-γ (mIU/mL) Median (IQR)
None	8	4953 (4245.5–11,280)	6	2425 (1144–3429)
One infection	145	14,359 ** (9318–22,008)	45	1815 (1038–2646)
Reinfection (2 or more infections)	76	13,143 ** (9413–17,654.5)	27	2944 * (1614–3863)

Statistically significant difference: * (<0.05), or ** (<0.01)). The Mann–Whitney U test was used to calculate *p*-values.

**Table 4:** Cellular immune response in non-infected, primoinfected, and reinfected individuals at different time points (only participants who received the fourth dose are included) (n = 229).

	Infecting Variant	N	IFN-γ (mIU/mL) Median (IQR)	*p*-Value
Primoinfection before December 2021	Wuhan, Alpha, Delta [14,15]	-	-	-
Primoinfection January 2022	Omicron (BA.1) [14,15]	9	1761 (508–2416)	NS
Reinfection January 2022	Omicron (BA.1) [14,15]	1	3309 (-)	
Primoinfection January–June 2022	Omicron (BA.1, BA.2) [14,15]	25	1949 (920–2646)	NS
Reinfection January–June 2022	Omicron (BA.1, BA.2) [14,15]	5	2137 (2038–2820)	
Primoinfection July 2022–October 2023	Omicron (BA.5, BQ.1, XBB.1.5) [16]	11	1787 (1424–3634)	NS
Reinfection (1 or more) July 2022–October 2023	Omicron (BA.5, BQ.1, XBB.1.5) [16]	21	2976 (1457–4247)	
Non infected	-	6	2425 (1144–3429)	-

NS: non-significant. The Mann–Whitney U test was used to calculate *p*-values.

**Table 5:** Anti-S-RBD levels considering low or high levels of cellular immunity (n = 169).

	N	Anti-S-RBD (U/mL) Median (IQR)
Low level IFN-γ (mIU/mL)	53	13,183 (5629–20,442)
High level IFN-γ (mIU/mL)	116	15,830.5 (9414.5–22,701.5)

**Table 6:** Humoral and cellular immune response by initial vaccination schedule (n = 549).

Initial Vaccine Schedule	N	Anti-S-RBD (U/mL) Median (IQR)	N	IFN-γ (mIU/mL) Median (IQR)
ChAdOx1 single dose	25	9275 (5218–10,529)	7	1919 (1323–2137)
ChAdOx1/ChAdOx1	124	10,353 (5417–14,156)	29	2068 (1424–3240)
mRNA-1273/mRNA-1273	229	14,447 *** (9513–22,008)	68	2395.5 (1142–3618.5)
BNT162b2/BNT162b2	123	14,868 *** (9162–22,044)	56	2328.5 (1459.5–3339.5)
ChAdOx1/BNT162b2	48	14,639 ** (8473.5–21,258.5)	14	1416.5 (1043–2222)

Statistically significant difference: ** (<0.01), or *** (<0.001). The Mann–Whitney U test was used to calculate *p*-values.

## Data Availability

The data that support the findings of this study are openly available in the Harvard Dataverse at https://doi.org/10.7910/DVN/3HRDXL (accessed on 9 December 2024).

## Supplementary Materials

The following supporting information can be downloaded at: https://www.mdpi.com/article/10.3390/vaccines12121408/s1, Table S1: Humoral immune response (Anti-S-RBD (U/mL) median (IQR)) by age and sex (n = 549); Table S2: Cellular immune response (IFN-γ (mIU/mL) median (IQR)) by age and sex (n = 174).
