# Supplementary material for: Impact of Repeated Variant Exposures on Cellular and Humoral Immunogenicity Induced by SARS-CoV-2 Vaccines
Source: Vaccines (Basel). 2024 Dec 13;12(12):1408. doi: 10.3390/vaccines12121408 (PMC11680034; doi:10.3390/vaccines12121408)
Supplement: Supplementary file 1 [file vaccines-12-01408-s001.zip › vaccines-3342055-supplementary.pdf]

SUPPLEMENTARY MATERIAL

Table S1. Humoral immune response (Anti-S-RBD (U/mL) median (IQR)) by age and sex (n=549).

	N	4th dose (n=229)	No 4th dose (n=320)	TOTAL (n=549)
**Women (n)**	483	13393 (9311-20969) (199)	11785 (6589-20075) (284)	12727 (8083-20245)
**Men (n)**	66	13577 (8986-22044) (30)	11365 (9229-20484) (36)	13034.5 (9068-20977)
**<=50 years (n)**	307	11754 (8003-18213) (113)	11294 (7085-16745) (194)	11392 (7185-17180)
**>50 years (n)**	242	14105** (10433-24734) (116)	16115** (8745-29292) (126)	14317*** (9275-28152)

Statistically significant difference: * (<0.05), ** (<0.01), or *** (<0.001). The Mann-Whitney U test was used to calculate p-values.

Table S2. Cellular immune response (IFN-γ (mIU/mL) median (IQR)) by age and sex (n=174).

	N	4th dose (n=78)	No 4th dose (n=96)	TOTAL
**Women (n)**	145	2259 (1181-3270) (64)	2068 (1199-3321) (81)	2117 (1199-3309)
**Men (n)**	29	1825 (1138-2499) (14)	3136** (2558-4695) (15)	2558 (1707-3453)
**<=50 years (n)**	95	1761 (1146-2976) (35)	2188 (1542-3529) (60)	2068 (1325-3231)
**>50 years (n)**	79	2316 (1360-3309) (43)	2202 (1045-4187) (36)	2297 (1122-3453)

Statistically significant difference: * (<0.05), ** (<0.01), or *** (<0.001). The Mann-Whitney U test was used to calculate p-values.
